# Understanding the Mode and Factors Influencing Cut-Throat Injuries in a Tribal-Dominated Population in Eastern India

**DOI:** 10.7759/cureus.45481

**Published:** 2023-09-18

**Authors:** Md Anas, Raihan Mannan, Zahid M Khan

**Affiliations:** 1 Otolaryngology, Rajendra Institute of Medical Sciences, Ranchi, IND; 2 Physiology, All India Institute of Medical Sciences, Patna, Patna, IND

**Keywords:** tribal community, substance abuse, suicide, homicide, cut-throat injury

## Abstract

Introduction

Traditionally, injuries have often been perceived as random and inevitable 'accidents'. However, in the current context, both intentional and unintentional injuries are preventable. Among these, neck injuries can be particularly complex, encompassing homicidal, suicidal, or accidental causes. Despite extensive research on the different modes and patterns of cut-throat injuries in the general population, this study aims to look deeper into this phenomenon within a unique context. Our investigation is centered in the Eastern part of India, among a tribal-dominated population who live with their traditional culture.

Method

In this prospective observational study, 50 cut-throat patients were included, who reported to the emergency department and then were referred to the ENT department. Parameters such as age, sex, anatomical location, mode of cut-throat injury, prior substance abuse, psychiatric issues, socioeconomic level, and length of hospital stay were considered for analysis.

Results

The majority (n=40, 80%) of patients belonged to tribal communities and most were young adults. Cut-throat injuries were predominantly caused by suicide (n=36, 72%) and homicide (n=12, 24%). Among tribals, 85% (n=34) of the cases were suicidal. Among suicidal cases, 86.11% (n=31) had a history of substance abuse while 22.22% (n=8) had a history of psychiatric illnesses.

Conclusion

In Eastern India, among the tribal-dominated population, suicide emerges as the predominant mode of cut-throat injuries, which is notably distinct from patterns observed in the general population of developing countries. These were often associated with substance abuse. Future interventions and public health efforts in these regions should consider these factors in the development of targeted strategies aimed at prevention and support for at-risk individuals.

## Introduction

According to WHO Global Health Estimates-2019, suicide is the fourth leading cause of death in young people aged 15-29 years, after road injury, tuberculosis, and interpersonal violence [1]. Approximately, 8,00,000 people die by suicide worldwide every year, with the suicide rate being 2.3 times higher in males than in females. Suicide is an emerging and serious public health issue in India.

Homicide, suicide, and accident are possible modes of cut-throat injury, which refers to an open injury to the neck. These wounds can be caused by knives, razor blades, broken glass, gunshots, and punctures. Neck injuries frequently pose a life-threatening and require rapid medical attention [2]. The extent of neck injuries can be determined by assessing the structures involved, for which the Roon and Christensen classification provides a useful framework [3].

Globally, 5-10% of all traumatic injuries are slit throats, and in 30% of cases, multiple structures are affected [2-4]. In developing countries like India, illiteracy, lack of employment, poverty, drug and alcohol misuse, and low socioeconomic conditions contribute to increased rates of crime and suicide. Escalating conflicts over limited resources are also contributing factors. Asphyxia, hemorrhage, and shock are the main causes of death in cut-throat injuries. The management of cut-throat injuries requires a multidisciplinary approach involving otolaryngologists, vascular surgeons, anesthesiologists, and psychiatrists [4].

In developed nations, suicide is the most frequent cause of cut-throat injuries, whereas, in developing countries like India, suicidal cut-throat injuries are rare, with homicidal cases being more common [5,6]. Recent studies in India have reported similar findings [7]. The present study was conducted in Jharkhand, a region of Eastern India, where approximately 26% of the state’s population consists of tribal people. The study aimed to investigate the frequency of different modes of cut-throat injury (homicidal, suicidal, and accidental) and the pattern (zones and extents) of cut-throat injuries among the tribal-dominated population. Furthermore, it aimed to determine other influencing factors, including age, sex, substance abuse, psychiatric illness, and socioeconomic status.

## Materials and methods

This prospective observational study was conducted at a tertiary care hospital, Rajendra Institute of Medical Sciences, Ranchi, Jharkhand, in Eastern India. A total of 50 cut-throat patients presented to the emergency department and the ENT department from January 1, 2020, to June 30, 2022, were included in this study. Patients between the ages of 10-80 years were enrolled, and consent was obtained either from the patients or from their parents/guardians in the case of minors. Unconscious patients, those who refused consent, and those with associated head injuries were excluded from the study.

Age, sex, anatomical location and mode of cut-throat injury, substance abuse, psychiatric issues, socioeconomic level, and length of hospital stay were the parameters considered for data analysis. The depth and anatomical location of neck injuries were determined by assessing the structures involved, for which the Roon and Christensen classification was used [3]. For the assessment of mental status, each patient was consulted by the psychiatry department. The Modified Kuppuswamy socioeconomic scale, based on occupation, education, and monthly family income, was used to determine the socioeconomic category [8]. The study protocol and procedures were approved by the Institutional Ethics Committee of Rajendra Institute of Medical Sciences (RIMS), Ranchi (approval number: 209IEC, RIMS).

Evaluation and management of a patient with a cut-throat injury

The evaluation of cut-throat injuries followed the Advanced Trauma Life Support (ATLS) guidelines, with the first primary survey emphasizing the examination of the airway, breathing, and circulation. In cases of compromised airway, a tracheostomy was performed to secure the airway. After stabilizing the patient, an essential secondary survey was conducted, involving a comprehensive physical examination and detailed medical history, along with relevant investigations.

The wound was cleansed thoroughly using saline, followed by an antibiotic solution and betadine. Antibiotics and tetanus toxoids were administered, and the extent of the wound was examined for injuries to the pharynx, larynx, and trachea.

Closure of surgical wound

Surgical wound closure involved layer-by-layer suturing of the superficial cut-throat wound using 2-0 or 3-0 vicryl under local anesthesia. In cases of deep injuries to the pharynx, larynx, or trachea, a tracheostomy was performed under general anesthesia. Vicryl 3-0 was used to repair the soft tissue's mucous membrane and muscles while proline 2-0 was used for wound closure. Ryles tubes were inserted in patients with laryngeal and pharyngeal injuries.

Statistical analysis

Descriptive analysis was done to provide a comprehensive characterization of the patient population. Additionally, frequency analysis was conducted to determine the distribution and proportions of categorical variables, including age, gender, depth and anatomical sites of cut-throat injuries, mode of cut-throat injury, mental status, substance abuse, socioeconomic class, as well as the duration of hospital stay. All the data were entered and analyzed using Google Sheets (Google LLC, Mountain View, CA).

## Results

Patient characteristics

In total, 50 patients with cut-throat injuries were included in our study. Out of which, 40 (80%) patients belonged to the tribal population and 10 (20%) patients belonged to the non-tribal population. The male: female ratio was 11.5: 1, 46 were male, and four were female. The ages ranged from 10 to 80 years, and the commonest age group affected was 21-40 years (Table 1).

**Table 1:** Patient characteristics (n=50)

Characteristics	Number	Percentage (%)
Tribal patients	40	80
Non-tribal patients	10	20
Male	46	92
Female	4	8
Male: Female ratio	11.5:1	

Age and gender distribution

**Figure 1:**
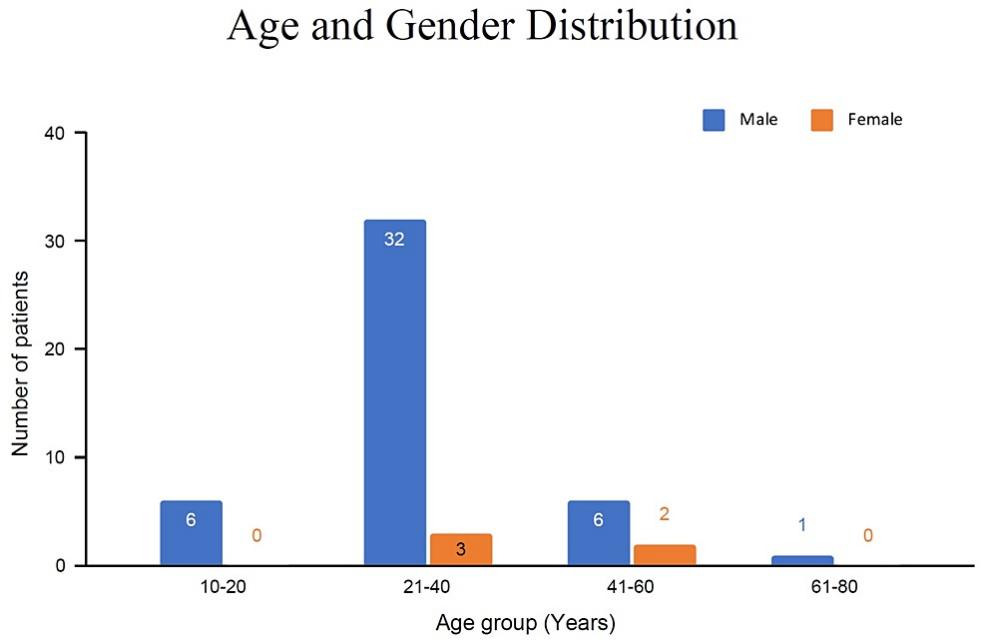
Age and gender distribution of cut-throat patients

Patients between the ages of 21-40 made up the majority (70%) of the population, with 32 males and three females, followed by age group 41-60 (16%), six males and two females. Only one was above 60 years of age and six were below 20 years of age and these age groups comprised male patients only (Figure 1).

Depth and anatomical sites (zones) of cut-throat injuries

**Figure 2:**
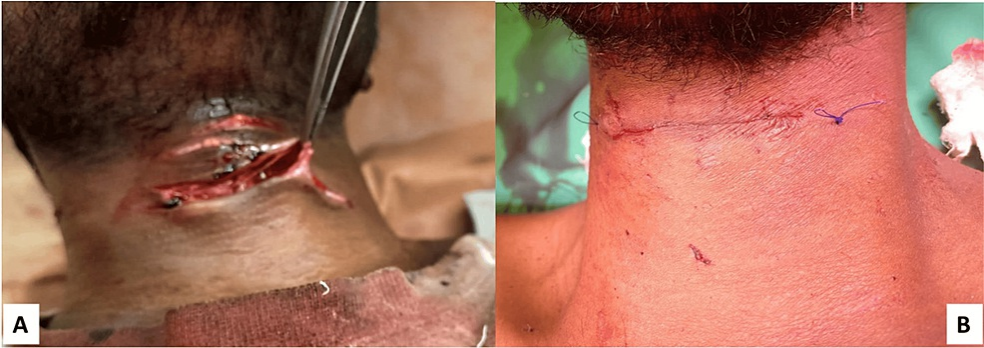
Preoperative (A) and postoperative (B) picture of a suicidal cut-throat patient having a superficial injury

**Figure 3:**
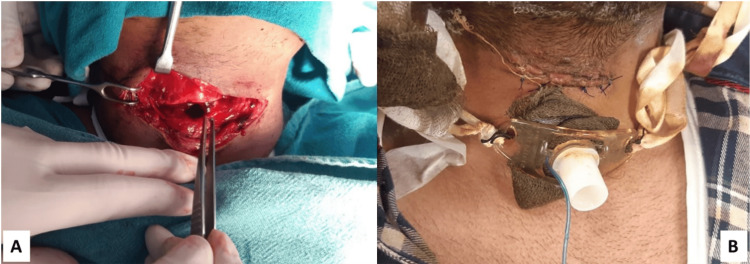
Preoperative (A) and postoperative (B) picture of a suicidal cut-throat patient having a deep injury with a breach of the airway A tracheostomy was done to secure the airway.

The superficial injuries were managed with a simple layer-by-layer closure of the wound under local anesthesia (Figure 2). The deep injuries were managed under general anesthesia. A tracheostomy was performed to secure the airway, and the wound was closed in two layers (Figure 3).

According to anatomical classification, no patient was in Zone I, 38 (76%) patients were in Zone II, and 12 (24%) patients were in Zone III (Table 2).

**Table 2:** Depth and anatomical sites of cut-throat injuries

Zone of Injury	Number of patients	Percentage (%)
Zone I	0	0
Zone II	38	76
Zone III	12	24

Mode of cut-throat injury

**Figure 4:**
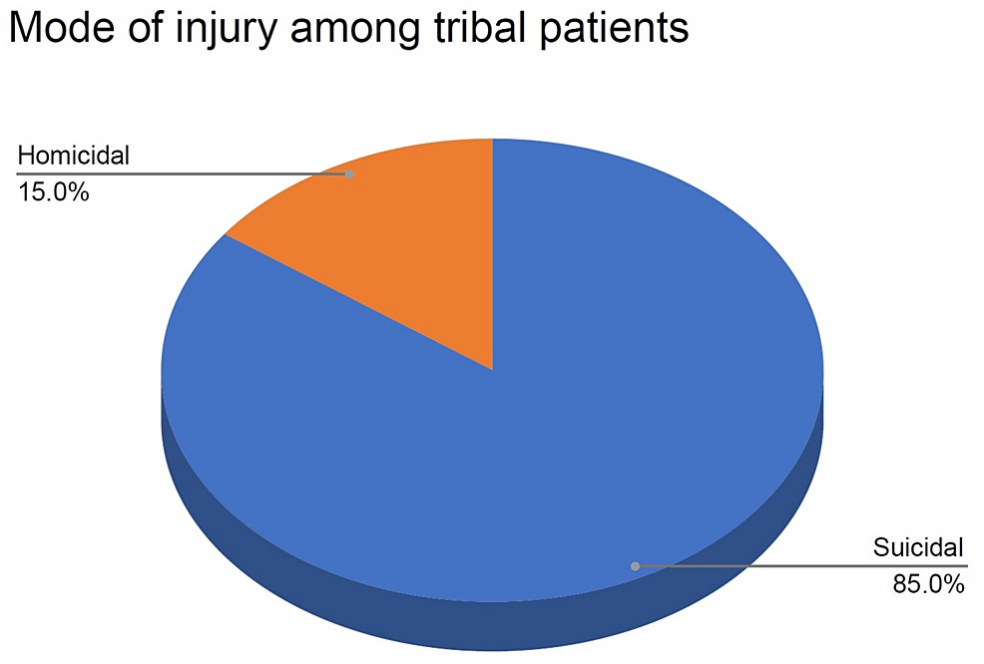
Percentage of the mode of injuries in the tribal population

Among various modes of cut-throat injury, in this study, suicidal cases were high constituting 72% (n=36), followed by homicidal cases, which were 24% (n=12) of cases, and then accidental cases, which were 4% (n=2), as shown in Table 3. Among tribal patients, the suicidal mode of injury accounted for 85% (n=34) of tribal cases, whereas, the homicidal mode of injury accounted for only 15% (n=6) (Figure 4).

**Table 3:** Modes of cut-throat injury cases

	Suicidal		Homicidal		Accidental	
	Number	Percentage (%)	Number	Percentage (%)	Number	Percentage (%)
Tribal	34	85	6	15	0	0
Non-tribal	2	20	6	60	2	20
Total	36	72	12	24	2	4

Mental status and substance abuse

Psychiatric consultation was undertaken for every patient to evaluate mental illness. Eighty-six point eleven (86.11%; n=31) of the suicidal cut-throat injury cases were found to be addicted to locally accessible drugs, and 22.22% (n=8) of the suicidal cases had psychiatric problems. The most often misused substances were cannabis, nicotine, and home-brewed alcoholic beverages, including hadiya rice beer and mahua liquor derived from mahua flowers (Table 4).

**Table 4:** Risk factors for suicidal cut-throat injury cases (n=36)

Types of risk factors	Number of patients	Percentage (%)
Substance abuse	31	86.11
Psychiatric illness	8	22.22

Socioeconomic class

In addition to substance abuse and psychiatric illness, other risk factors significant for suicidal mode of cut-throat injury include illiteracy, unemployment, and poverty. The majority (56%) of patients in this study belonged to the lower middle class (Table 5).

**Table 5:** Socioeconomic class-wise distribution of cut-throat injury patients

Socioeconomic class	Number of patients	Percentage (%)
Upper	0	0
Upper middle	0	0
Middle	16	32
Lower middle	28	56
Lower	6	12

Duration of hospital stay

The average hospital stay was 15 days. Fifty-two percent (52%) of the cut-throat injury patients stayed in the hospital for 10-20 days, 42% of the patients stayed for less than 10 days, and only 6% of the cases stayed for more than 20 days. Infection of the wound, voice changes, and restricted neck mobility were the most frequent causes of longer hospital stays (Table 6).

**Table 6:** Days of hospital stay

Days	Number of patients	Percentage (%)
< 10	21	42
10-20	26	52
> 20	3	6

## Discussion

In this prospective study, we aimed to investigate the frequency and prevalence of different modes of cut-throat injury (homicidal, suicidal, and accidental) and the pattern (zones and extents) of cut-throat injuries in a tribal-dominated population in Eastern India. Our findings showed that 80% of the patients belonged to the tribal community, and the male-to-female ratio was 11.5:1. Most of the patients (72%) were adults in their third or fourth decades of life, which aligns with previous studies. For instance, Nason et al. reported 130 cases of cut-throat injuries with ages ranging from 4 to 74, comprising 109 male and 21 female cases [9]. 

Regarding the mode of cut-throat injury, our study revealed a high prevalence of suicide cases (72%) followed by homicidal cases (24%). Among tribal patients specifically, the suicidal mode of cut-throat injury accounted for 85% (n=34) of cases while the homicidal mode accounted for only 15% (n=6). This finding contrasts with previous studies in the general population of developing countries, where homicide was the most common cause of cut-throat injuries [6]. Conversely, in developed nations, suicide has been reported as the most frequent reason [5]. Globally, suicide is the second-leading cause of death for individuals aged 15 to 24 [10].

Zone II injuries were the most prevalent in our study, similar to findings from previous studies. Sriussadaporn et al. explained that Zone II is more vulnerable to neck trauma than Zones I and III due to its lack of bone protection [11]. Another study by Mahmoodie et al. reported that Zone II accounted for 56.3% of penetrating neck injuries [12].

In our study, we identified self-inflicted cutting wounds as a common method of suicide. Psychosocial factors and substance abuse were frequently found in cases involving the suicidal mode of cut-throat injuries. Specifically, alcohol misuse stood out as a prominent form of substance abuse. According to the National Family Health Survey 2020-21 (NFHS-5), alcohol addiction is a major public health concern in the state of Jharkhand, with stress, unemployment, and poverty being the main causes of substance abuse. Locally produced alcohol such as mahua and hadia are easily accessible to the local population. Mahua, derived from the Madhuca longifolia flower, holds cultural significance among indigenous tribes living in the forest. The overconsumption of these alcoholic beverages leads to stress, mental instability, physical illness, and ultimately, suicidal attempts. These issues are compounded by the underlying causes of illiteracy, unemployment, poverty, and lack of alternative leisure activities.

Regarding treatment, patients frequently underwent procedures such as tracheostomy, laryngeal repair, hypopharyngeal repair, and primary wound closure. The average hospital stay was 15 days, which aligns with a study conducted by Aich et al., where the majority (73.13%) of patients were discharged within 14 days [13].

Limitations of the study

Despite having the largest sample size in this field to date, our study has several limitations. The sample size, while extensive, remains relatively small in the context of epidemiological research. We relied on patient and attendant-provided information, potentially introducing inaccuracies, especially in reporting substance abuse and addiction. Furthermore, the generalizability of our findings to other regions or populations may be limited. Inherent biases as well as the potential impact of cultural factors should also be considered.

## Conclusions

Contrary to previous studies in the general population of developing countries, which often reported homicide as the leading cause of cut-throat injuries, our study revealed that suicide was the primary reason for such injuries among adults in the tribal-dominated population in Eastern India. Uneducated, unemployed individuals from lower socioeconomic levels, who were also abusing one or more drugs, were more susceptible. Addressing the root problems, such as illiteracy, unemployment, poverty, psychiatric illnesses, and substance abuse, will be crucial in reducing the incidence of cut-throat injuries. Future interventions and public health efforts in these regions should consider these factors in the development of targeted strategies aimed at prevention and support for at-risk individuals.
